# Utilization of antiretroviral treatment in Ethiopia between February and December 2006: spatial, temporal, and demographic patterns

**DOI:** 10.1186/1476-072X-6-45

**Published:** 2007-09-25

**Authors:** Helmut Kloos, Yibeltal Assefa, Aynalem Adugna, Mesfin Samuel Mulatu, Damen Haile Mariam

**Affiliations:** 1 Department of Epidemiology and Biostatistics, University of California, San Francisco Medical Center, San Francisco, USA; 2 Ethiopian HIV/AIDS Prevention and Control Office, Ministry of Health, Addis Ababa, Ethiopia; 3 Department of Geography, Sonoma State University, Sonoma, CA, USA; 4 Center for Community Prevention and Treatment Research, The MayaTech Corporation, Atlanta, USA; 5 Department of Community Health, Addis Ababa University, Addis Ababa, Ethiopia

## Abstract

**Background:**

In 2003, the Ethiopian Ministry of Health (MOH) started to implement a national antiretroviral treatment (ART) program. Using data in the monthly HIV/AIDS Updates issued by the MOH, this paper examines the spatial and temporal distribution of ART on a population basis for Ethiopian towns and administrative zones and regions for the period February to December 2006.

**Results:**

The 101 public ART hospitals treated 44,446 patients and the 91 ART health centers treated 1,599 patients in December 2006. The number of patients currently receiving ART doubled between February and December 2006 and the number of female patients aged 15 years and older surpassed male patients, apparently due to increased awareness and provision of free ART. Of 58,405 patients who ever started ART in December 2006, 46,045 (78.8%) were adhering to treatment during that month. Population coverage of ART was highest in the three urban administrative regions of Addis Ababa, Harari and Dire Dawa, in regional centers with referral hospitals, and in several small road side towns that had former mission or other NGO-operated hospitals. Hospitals in Addis Ababa had the largest patient loads (on average 850 patients) and those in SNNPR (Southern Nations and Nationalities Peoples Republic) (212 patients) and Somali (130 patients) regions the fewest patients. In bivariate tests, number of patients receiving treatment was significantly correlated with population size of towns, urban population per zone, number of hospitals per zone, and duration of ART services in 2006 (all p < 0.001). The stronger relationship with urban than total zonal populations (p < 0.001 versus p = 0.014) and the positive correlation between distance from 44 health centers to the nearest ART hospital and patients receiving treatment at these health centers may be due to a combination of differential accessibility of ART sites, patient knowledge and health-seeking behavior.

**Conclusion:**

The sharp increase in ART uptake in 2006 is largely due to the rapid increase in the provision of free treatment at more sites. The marked variation in ART utilization patterns between urban and rural communities and among zones and regions requires further studies. Recommendations are made for further expansion and sustainability of the ART scale-up.

## Background

Although the number of new HIV infections worldwide still outpaced the expansion of antiretroviral treatment (ART) for HIV infection in 2006, major inroads have been made in recent years in developing countries. The Global Fund to Fight AIDS, Tuberculosis and Malaria (Global Fund) made generic antiretrovirals (ARVs) eligible for funding in 2002, drastically reducing the price of these drugs and promoted the development of simpler fixed-dose combination therapies [1]. The 3 × 5 Initiative of WHO gave early impetus to securing resources for treatment and led to a near-doubling of people put on treatment. The U.S. President's Emergency Plan for AIDS Relief (PEPFAR), in collaboration with the Global Fund to Fight AIDS, Tuberculosis and Malaria (Global Fund) programs supported major scale-up of programs in many countries [1], and they are the two largest donors in the HIV/AIDS sector in Ethiopia [2]. Ethiopia, one of PEPFAR's 15 focus countries, received nearly $500 million between 2003 and 2006 from PEPFAR and the Global Fund [3,4]. In 2006, rapid expansion of ART services in health centers was pursued to increase access to treatment in rural areas [5]. Under the guidance of the Strategic Framework of the National Response to HIV/AIDS in Ethiopia for 2001–2005, and the Road Map 2004–2006, the ART rollout plan is being implemented with support from government sectors, NGOs, the private sector, faith-based organizations and the communities [6]. They use the public health approach advocated by WHO, which emphasizes standardized, simplified treatment protocols and decentralized service delivery involving mid-level health professionals [7]. Ethiopia has made significant progress in the recruitment and processing of eligible ART patients for referral by urban associations (*kebeles*) and community health workers, and in the provision of home-based health care by faith-based organizations and traditional burial societies (*iddir*) using nurses [8-10]. Also a M.Sc. program in Health Monitoring and Evaluation has recently been launched to reduce manpower shortage [11]. The ambitious ART plan of the Ethiopian government called for 100,000 of the estimated 286,258 people living with HIV/AIDS (PLWHA) in need of continuous, lifelong treatment to be put on ART by the end of 2006 [6].

According to the Sixth *AIDS Report *by the MOH, 3.5% of all Ethiopians (10.5% of the urban population aged 15–49 and 1.9% of the rural population) were infected with HIV in 2005 [12]. These data were updated for 2006 by incorporating the lower rates of the 2005 Ethiopia Demographic and Health Survey and published as single point prevalence of 2.1% [13]. The Ministry of Health estimated that an estimated 929,699  people in Ethiopia lived with HIV/AIDS in 2006. Modeling of infection rates and VCT data suggest that the epidemic is continuing to expand throughout Ethiopia except in some urban areas [13,14]. In response to the HIV/AIDS epidemic, the Ethiopian government issued policies and developed programs for prevention of HIV since the mid 1980s and in 2003 started an ART program in the largest urban centers, which has since then been extended to smaller towns and rural communities in all 11 regions. In view of the non-affordability of ART by most HIV-infected persons in Ethiopia, the MOH launched the free ART rollout program in January 2005, a strategy which has been associated with increased access to ART and lower mortality in other developing countries [15]. By mid 2006, health centers in different regions, staffed by newly trained health officers and nurses, started to provide treatment as part of up-scaling ART [6,12].

Existing information on the geographic distribution of AIDS patients treated in urban health facilities shows that in 1990, when all health facilities providing palliative treatment to AIDS patients were still in Addis Ababa, 87.6% of the total of 636 patients that were reported to the MOH lived in this city and that the male/female ratio was 2.3 to 1 [16]. By the end of July, 2006, 35,460 patients were currently receiving ART at 93 health facilities in all 11 regions. Nearly half of the patients (43.2%) were recorded at facilities in Addis Ababa. Of these, 47% were males above 14 years and 48% females, with 4% children [12]. The proportion of patients from Addis Ababa was actually smaller because of the influx of patients with AIDS and other diseases from other towns and rural areas seeking better treatment or less stigma [17,18]. Utilization data of the monthly HIV Care and ART Updates of the Ministry of Health [19] also indicate considerable spatial variation but they have not been analyzed.

As part of implementing the ART rollout, the MOH issued regional targets and ARV drug quotas for each region based on the estimated number of PLWHA eligible for ART and geographical and health services parameters. It proposed that catchments areas of individual health facilities be mapped in an effort to facilitate the equitable distribution of ART [6]. An earlier study on the size of catchments areas for polyclinic outpatients and inpatients in different parts of the country showed that they range from *wereda *(district) sized areas for health centers to entire regions for well established hospitals [20]. The three major databases on the geographic distribution of HIV infection throughout Ethiopia, an important factor in the demand for ART services, are deficient due to data aggregation at the regional level and their restriction to young female antenatal attendees and male army recruits [12,13,21]. The geographic distribution of ART has not been studied. The objective of this study is to provide a preliminary analysis of the spatial and temporal distribution of ART utilization at the town, zonal and regional levels in relation to population distribution for the period February to December 2006 using monthly MOH treatment data. The results may identify spatial and demographic factors in promoting the comprehensive HIV/AIDS care approach, which focuses on the catchments area as the major geographic unit of program implementation for individual ART sites [6].

## Methods

### Data

ART data in the monthly HIV/AIDS updates prepared by the Ethiopian AIDS Resources Center (Etharc) for 2006, covering the period February-December 2006 [19] and all ART treatment centers except those serving in the military were analyzed in relation to demographic and geographic variables. Military treatment centers were excluded to avoid geographical bias. The patient data analyzed here are categorized the same way as by Etharc into the three initial stages or phases of the HIV/AIDS treatment process: 1) enrollment for HIV/AIDS care, 2) starting on ART and 3) continuing treatment and currently on ART. Town and regional population data are based on the region-specific projections for 2006 by the 1994 national census [22]. These denominator populations were used to calculate ART utilization rates for urban areas, zones, and regions for December 2006, the month when the largest number of treatment facilities were treating AIDS patients. Treatment centers were identified on the 1:2,000,000 topographic map of the Ethiopian Mapping Agency and the ART maps prepared by Etharc for the preparation of population and ART utilization maps. Distances between towns with health centers lacking hospitals and towns with ART hospitals were obtained from the list prepared by the Ministry of Health [23]. Data on the number of patients treated in each hospital and health center can be accessed from the Etharc website [24]. Data on the 2006 projected population of towns are available from the authors upon request.

The requirements, criteria and procedures for enrollment for free ART include the following: HIV test results, personal and behavioral eligibility as it has an effect on adherence to treatment, some laboratory investigations, including hematology, CD4 counts and blood chemistry to determine the eligibility for and safety of ART. TB patients with CD4 counts less than 50 cells per ml need to be treated for TB for the first two months and then they can be started on ART [25]. Shortly after the launching of the free ART scheme, all patients were requested to bring a poverty certificate for free ART from their *kebele *administration office which also verified patients' place of residence. But later on this proved to be stigmatizing and was a barrier for patients to get treatment at the health facilities. Therefore, this requirement was abolished and currently ART is free throughout the country for all patients who need the treatment and are medically eligible for the treatment. VCT (voluntary counseling and testing) constitutes an integral part of the enrollment process, as described in the Ministry of Health Guidelines for Use of Antiretroviral Drugs in Ethiopia to promote adherence, confidentiality, and destigmatization [26]. The demographic, socioeconomic, and behavioral data collected during VCT and the clinical and anthropometric data obtained during pre-initiation may be used for subsequent analyses examining their relationship with initiation and adherence.

### Analysis

The Adobe Photo Delux Business Edition software was used in conjunction with MS Excel for mapping and the SPPS program version 12.0 for statistical analysis. This preliminary study uses mostly descriptive analysis.

## Results

### Distribution of ART sites and their patient loads

**Figure 1:**
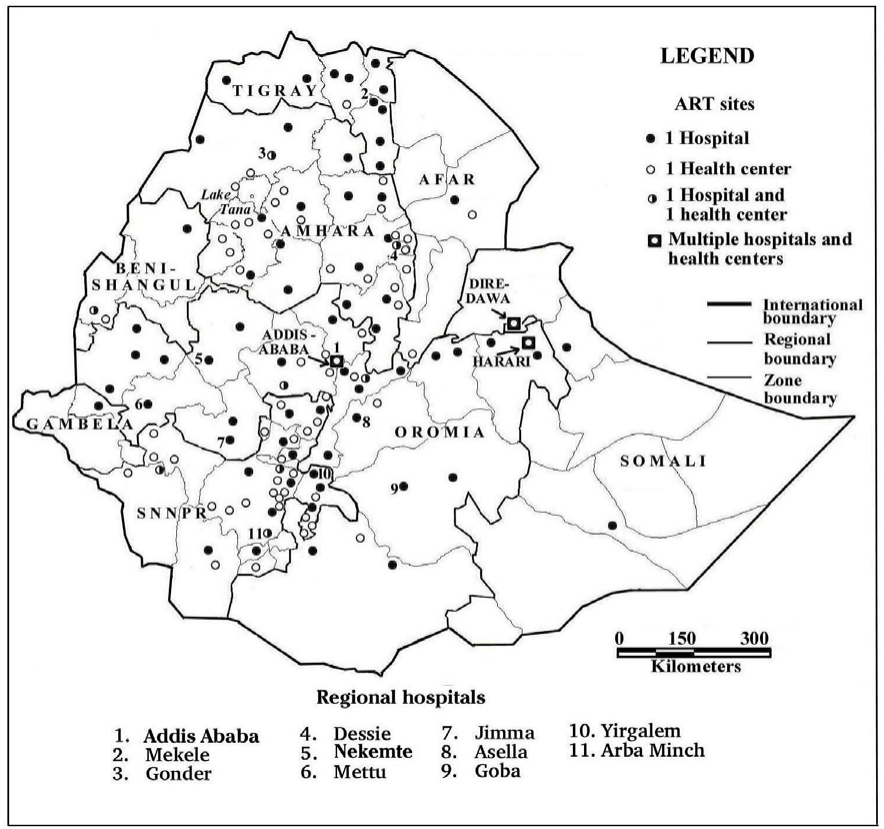
The 136 towns providing ART in December 2006, by type of facility, region and zones.

By December 2006, a total of 96,897 AIDS patients had ever been enrolled at 192 ART facilities, 58,405 had ever started ART and 46,045 were currently receiving ART at 168 facilities, constituting a drop-out rate of 20.7% for patients who had ever started ART. The actual treatment adherence/retention rate (78.8%) was higher because deaths were included in the default data. Including military personnel, 48,198 (98.9%) of all patients currently on ART in December 2006 were on a first line regimen of combination therapy consisting of stavudine/zidovudine, lamuvidine, and nevirapine/efavirenz. Only 121 (0.25%) patients were on a second line regimen, and 418 (0.86%) were on an unspecified regimen, indicating that nearly all recipients were classified as uncomplicated cases, although mortality probably prevented the documentation of more complicated cases. Documented uncomplicated cases comprise cases that don't require hospitalization and that have no other severe infections. The data for December 2006 were used to analyze and map the distribution of patients for towns, zones and regions where they obtained treatment. A total of 44,446 patients (96.5% of all patients nation-wide) received treatment from hospitals and only 1,599 (3.5%) patients obtained treatment from health centers during that month. Health facilities providing ART services were located in 140 towns, 134 of which had census population data and 136 of which could be mapped (Figure 1). The one hospital and six health centers located in seven small towns in SNNPR and Oromia without census data are unlikely to affect the statistical results as they served only 43 (0.1%) of all patients on ART in December 2006. The distribution of patients treated in individual hospitals and health centers was highly skewed toward larger, more established facilities that are better staffed and equipped. In December 2006, the 10 most utilized hospitals (the top 10%), treated 24,165 out of a total of 44,446 (54.4%) patients treated in hospitals during that month and the 10 least utilized hospitals served only 79 (0.2%) of the patients. Similarly, the top ranking 10 health centers served 637 of the 1,599 (39.9%) patients treated by health centers and 23 health centers treated no patients in December.

### Spatial and demographic patterns in the distribution of ART

**Figure 2:**
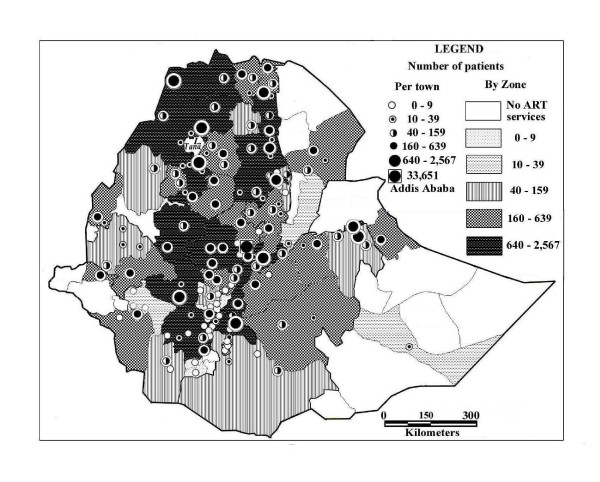
Number of patients receiving ART in 136 towns  and 47 zones in December 2006.

**Figure 3:**
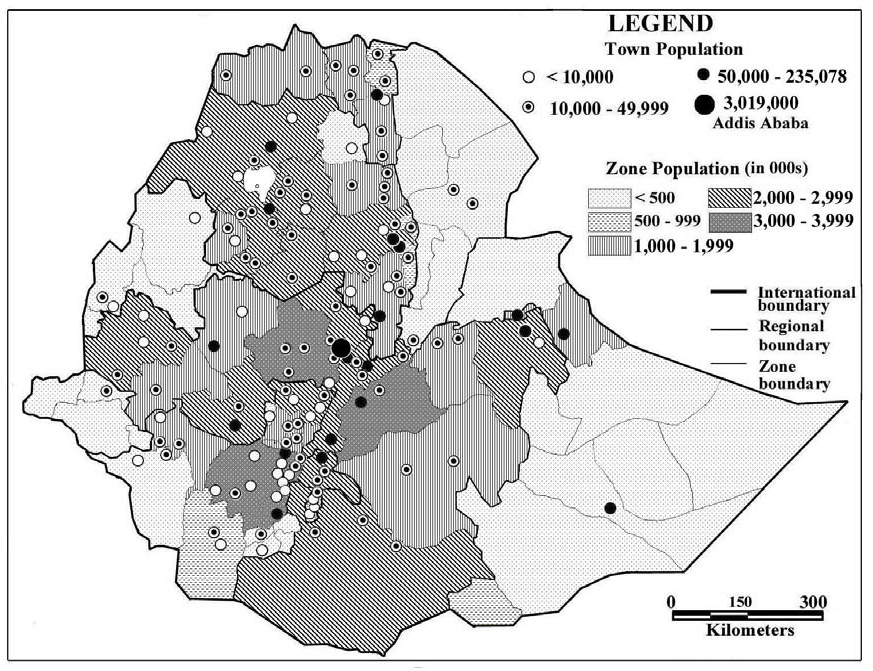
2006 projected population for 136 towns and 47 zones providing ART in December 2006.

A broad, continuous belt of urban ART sites serving more than 640 patients per town and zone in December 2006 extends in a north/south direction over the Ethiopian Highlands from western Tigray to SNNP Region, with a shorter north-south belt to the east of it in Tigray and Amhara regions (Figure 2). They comprise mostly large regional towns with referral hospitals, large zonal populations and are located on the Addis Ababa-Dessie-Mekele and the Gondar-Bahir Dar-Addis Ababa-Adama-Shashemene-Yirgalem-Arba Minch transportation routes, with east-west road connections to Jimma and Nekemte towns. The Dire Dawa and Harari city regions also had high utilization rates. Seventeen zones, nearly all of them in the peripheral lowlands, still had no ART services by December 2006 (figures 1,2,3). Percent urbanization and number of hospital beds per 10,000 population were broadly associated with number of patients who ever started ART by December 2006. A similar distribution of physician/population ratios was noted, ranging from 24.3 physicians per 100,000 population in Harari to 0.1 in Somali (Table 1).

**Table 1:** Population, no. and percentage of patients who started ART in relation to targets, percentage urbanization, number of hospital beds, and number of physicians, by administrative region

Region	2006 projected population *	No. of patients who started ART by December 2006	ART target for 100,000 patients for December 2006 **	Percent	Projected % urbanization for 2006 *	No. of hospital beds per 10,000 population in 2005 #	No. of physicians per 100,000 population in 2005 #
Addis Ababa	3012762	22291	16491	100	100	8.3	7.8
Harari	196000	951	1366	69.6	62.2	24.3	21.6
Dire Dawa	398000	1247	1114	100	74.4	9.2	7.8
Gambela	247000	235	912	25.8	19.0	3.9	2.5
Tigray	4335000	4471	6025	74.2	18.8	2.9	1.8
Benishangul	625000	544	1730	31.4	9.9	4.1	2.3
Amhara	19120000	13962	35448	39.4	11.5	0.8	0.7
Oromia	26553000	9711	22530	43.1	13.3	0.9	0.7
SNNPR	14902000	4197	12372	33.9	8.8	1.8	0.7
Afar	1389000	420	1108	37.9	9.1	0.9	1.2
Somali	4329000	376	3513	10.7	17.0	1.0	0.1
Total	75067000	58405	100000	58.4	16.2	1.8	2.2

* – source: based on [22]

** – source: based on [6]

# – source: based on [54]

**Figure 4:**
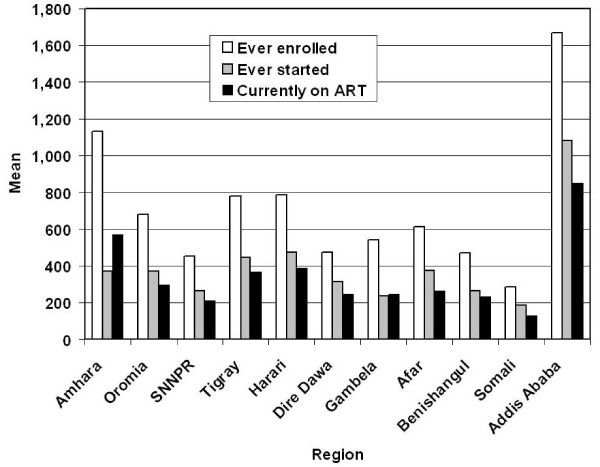
Number of patients ever enrolled, ever started, and currently on ART in 101 hospitals in December in 2006, by region.

**Figure 5:**
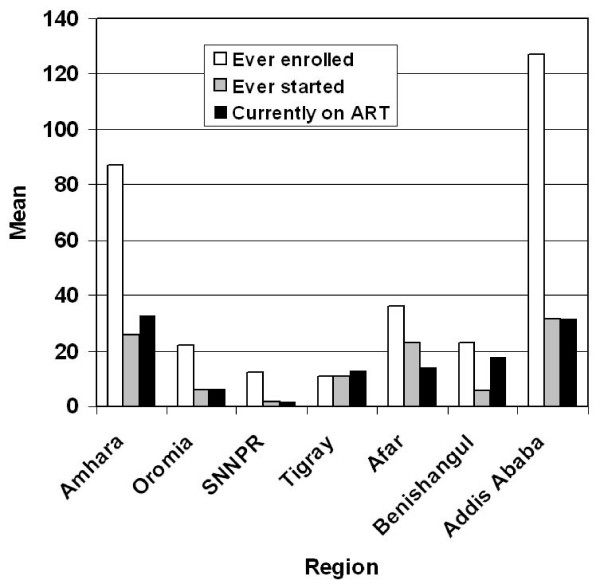
Number of patients ever enrolled, ever started, and currently on ART in 91 health centers in December 2006, by region.

The mean number of ART patients at each of the three stages of the treatment process varied considerably among ART sites in the different regions. The 21 hospitals in Addis Ababa had, on average, 850 patients currently on ART in December 2006, followed by those in Amhara (570), Harari (389), and Tigray (367). Hospitals in SNNPR (212) and Somali (130) had the fewest patients) [Fig. 4]. The largest number of patients on treatment during that month were reported by Zewditu Hospital (4,223) and ALERT Hospital (3,403), both in Addis Ababa, and both with a longer history of treating patients with major infectious diseases such as tuberculosis and leprosy. Individual health centers treated, on average, 17.6 patients during December 2006, ranging from 33 patients in Amhara Region and 31 in Addis Ababa to 1.6 in SNNPR [Figure 5]. The differences in mean numbers of both hospital and health center patients were statistically highly significant using the one-sample Kolmogorow-Smirnov test (p < 0.001). Follow-up studies analyzing utilization rates at the facility, town, zone and regional levels will also have to consider data on hospital size, ART capacity and number of patients from outside the study areas, which were not available to us.

The highest population-based ART rates among administrative regions were recorded for the city regions of Addis Ababa (0.59%), Harari (0.40%) and Dire Dawa (0.24%), all of which have multiple hospitals, and the smallest in facility-deficient SNNPR, Afar and Somali regions (0.006–0.02%). The regional distribution was similar to that of the 100,000 patients targeted for ART treatment in 2006 (Table 1).

At the town level, some of the highest ART rates (1.32% to 8.65%) were recorded for 9 small towns, all of them either with former mission or NGO health facilities or located on major transportation routes. Rates above 1.0% were also recorded in the following towns with regional or zonal hospitals: Dessie (1.54%), Bahir Dar (1.52%) and the district town of Woldia (2.03%) in Amhara Region, Mekele (1.06%) in Tigray Region, Mizan Teferi (1.37%) Yirgalem (2.55%) and Arba Minch (1.23%) in SNNPR, Adama (1.05%) in Oromia Region, and Assosa (1.03%) in Benishangul Region. All of them except Mekele and Woldia had 1 ART hospital and health center each in December 2006, and Dire Dawa and Harari had 4 and 2 hospitals, respectively (Figure 1). In Addis Ababa, 23 hospitals, including 10 public and 13 private hospitals as well as 24 public health centers served as ART sites in December 2006

The number of patients ever enrolled in chronic HIV/AIDS care, patients who ever started treatment, and patients currently receiving ART in December 2006 were highly correlated with projected 2006 populations of towns providing ART services (excluding Addis Ababa, Dire Dawa and Harari regions), as well as total urban populations of zones, number of hospitals per zone, and combined number of hospitals and health centers per zone (p < 0.001) and less so with zone population (p = 0.014) (Table 2). When the data on town populations were segregated by administrative region, the associations remained significant for all 4 regions with large sample sizes: Amhara, Oromia, Tigray and SNNPR (p < 0.02). Distance between the 44 health centers in towns without hospitals and the nearest ART hospital was positively but not significantly correlated with number of patients reported during the three treatment stages (Table 2).

**Table 2:** Bivariate correlations between patients currently on ART in December 2006 and demographic, treatment services and spatial variables

		**Pearson correlation**	**P**
**Population of towns providing ART**			
Mean	58150.4	0.985	<0.001
No.	133		
S.D. *	258455		
**Urban population per zone**			
Mean	252423	0.977	<0.001
No.	47		
S.D.	434850		
**Total population per zone**			
Mean	1477574		
No.	47	0.374	0.014
S.D.	969778		
**No. of hospitals per zone**			
Mean	2.1		
No.	47	0.948	<0.001
S.D.	3.2		
**No. of hospitals and health centers per zone**			
Mean	4.1		
No.	47	0.945	<0.001
S.D.	6.6		
Distance from health center to ART hospital **			
Mean	59.8		
No.	44	0.219	0.153
S.D.	33.6		
Duration of ART services in 2006 **			
Mean	6.2		
No.	192	0.476	<0.001
S.D.	3.7		

* – standard deviation

** – in km

# – in months

### Temporal patterns and drop-out rates

**Figure 6:**
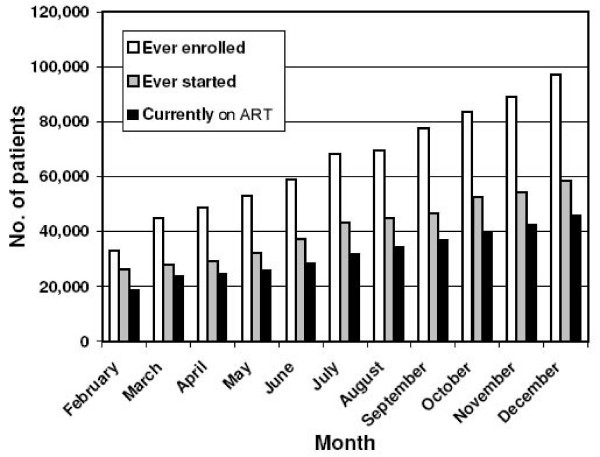
Patients ever enrolled, ever started on ART, and currently on ART, February to December 2006.

The total number of patients ever enrolled for chronic HIV/AIDS care at the 101 ART hospitals and 91 health centers increased from 41,935 in February 2006 to 96,897 in December 2006 (a 131.1% increase), that of patients ever started on ART increased from 25,356 to 58,405 (130.3% increase) and the number currently receiving treatment from 20,941 to 46,045 (119.9% increase) (Figure 6). Drop-out rates declined from 27.3% in February 2006 to 21.3% in December 2006, statistically significant in a chi-square test (p < 0.001). This decline was apparently due to a combination of a decrease in stigma and discrimination, an increase in awareness of the need for treatment adherence and the removal of the above-mentioned *kebele *screening criteria, although this requires further study. The drop-out rate includes both patients who defaulted and those who died, but the number of those who died is not indicated in the Etharc data set due to deficiencies in patient monitoring and related problems, inflating the drop-out rate.

Whereas 63 of the 68 hospitals operating in February 2006 had patients currently on ART during that month and all 101 ART hospitals by December, 3 of the 91 health centers started to provide ART in July and 23 of them (19 in SNNPR) still had no patients in December 2006. At the regional level, Addis Ababa Region ranked first in the increase in the number of patients currently on ART during that 11 month period, but the largest percentage increases were recorded in Gambela Region (276.9%), followed by Benishangul (214.7%), and the smallest increase occurred in Harari Region (72.9%). Overall, the largest percentage increases occurred in rural, peripheral, regions.

**Figure 7:**
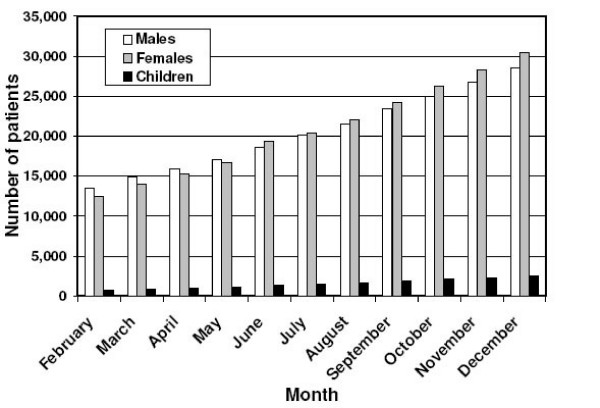
Infants and children below 15 years and males and females above14 years who started ART, February to December 2006.

The number of females aged 15 and older who ever started ART surpassed that of males in June 2006. Whereas 7.7% more males had ever started treatment in February 2006, by December 6.3% more females were in that category. Although the number of infants and children aged 5–14 who were being treated in December 2006 increased relatively faster (289%) than those for patients older than 14 years (233%) during this 11 month period, infants and children constituted only 4.7% of all patients on ART by December (Figure 7).

## Discussion

### Achievements, constraints and prospects in ART utilization

The number of ART hospitals serving the civilian Ethiopian population increased from 49 in late 2005 [5] to 67 in February 2006 and 101 in December 2006, and all 91 ART health centers became operational in mid 2006. A similar increase in the number of patients, relatively greater percentage increase in utilization in peripheral rural than urban regions, significant reduction in the drop-out rate, and a reversal of the male/female ratio in utilization, also reflect the rapid expansion of ART services and ART uptake nationwide. These achievements have been made possible primarily by the implementation of the ART rollout plan for 2004–2006, which implemented the scale-up of ART programs, the provision of free ART for all in need of treatment and generous outside funding. A total of 58,405 patients (58.4% of the 100,000 target) had ever started ART by December 2006 (Table 1), representing 23.9% of the 244,835 persons estimated to need ART in 2006 [13]. No specific information is available on the capacity of individual ART sites to treat more patients.

Further studies will be required to examine some of the above findings and also to address persisting concerns about accessibility to facilitate the scaling up and sustainability of ART. Thus the capacity of treatment sites, especially of health centers in the populous rural areas, to scale up ART and means to strengthen them needs further attention. The expansion of an upgraded health center ART network may contribute significantly to achieve patient targets [6] by overcoming lack of access to and awareness of ART sites, stigma, poor nutrition, high default and high mortality rates and a weak referral and linkage system [27]. More accurate drop-out rates may be determined with more complete mortality and treatment adherence data. A hospital-based study in SNNPR Region found that mortality from AIDS and TB is highest during the first few weeks after commencing ART [28,29]. In three hospitals in Addis Ababa, treatment adherence was found to be 81.2% among 431 patients in the week before the study [30]. The increase in ART utilization by females (Figure 5), who carried the greatest disease burden of HIV/AIDS in Ethiopia in 2006, has been attributed primarily to the provision of free ART since 2005 [29,31] because of gender-based inequitable access to financial resources, especially in rural areas [13]. An estimated 2.5% of females and 1.7% of males were infected in 2006 [13]. Increasing awareness of AIDS and the availability of free ART apparently were major factors in the to higher female ART uptake [12,27]. Nevertheless, female accessibility to ART remains low due to the location of treatment sites in hospitals and health centers and the stigma attached to seek treatment there, emphasizing the need for stronger community and home based care programs [32]. Although only slightly more than half of 4,000 children targeted for ART [6] were being treated by the end of 2006, the accelerating relative utilization rates for children below 15 years is an encouraging trend necessary to overcome underutilization due to inadequate access to ART by this age group [27].

The successful start of the Ethiopian ART program in 2003 and its rapid scale-up in 2006 in spite of inadequate health infrastructure, logistic problems, managerial problems and staff shortage is a remarkable achievement of the MOH and its partners. Demand and uptake by Ethiopians has not kept pace with the rapid expansion of ART services [33]. This discrepancy has prompted government, local NGOs and community-based organizations to implement comprehensive care programs at the community and households levels. By 2006, home and community-based programs that are enhancing the potential for AIDS patients to access needed services and that can facilitate prevention and stigma reduction activities were being implemented in 14 major towns [34]. Particularly effective in promoting pro-active HIV testing, treatment and other services is the collaborative community planning model by NASTAD, which was introduced in Ethiopia in 2002 and adapted for local use [35,36]. This bottom-up approach to HIV community planning is empowering communities to plan and implement preventive activities that support effective community mobilization, treatment adherence and wider uptake of services [33].

Various community-based programs which can compliment the institution-based approach pursued by the government, focus on community and home-based care and support [34,36,37]. They are being tried by various government and non-government agencies and include drug adherence follow-up [34], the recruitment of community-based reproductive health agents [38], the use of family care givers and case managers to improve treatment adherence [39], financial procurement and patient referral through the *iddir *and *mahiber *mutual help societies [10,40,41] and business-led responses [42]. Innovative approaches introduced in 55 hospitals in 2005 increased access to counseling and testing and doubled (from 21% to 44%) HIV-positive clients [43]. These various ART service related initiatives and programs, which are consistent with the public health approach advocated by WHO [8], will have to be dealt with in greater detail in follow-up studies.

In spite of these various achievements, it is not clear if the ART targets can be met or the scaling up of the program sustained in view of the persisting deficiencies of the health services, heavy donor dependence [44], possible donor fatigue [45], manpower shortage, high staff turn over and poor performance of health workers [44,46]. The current training and deployment of nurses in health centers, with the objective of shifting some of the clinical tasks such as initiating first-line ART for uncomplicated cases to mid-level health professionals is partly negated by heavy internal and external brain drain [6,43,47].

### Geographic and demographic patterns of utilization and implications for program development

Our analysis of the distribution of ART and population data at the zone and town levels reveals several patterns that can inform health planners on the accessibility of these services and in the delineation of hospital and health center catchments areas. The strong correlation between number of patients and population size at the community, zonal and regional levels (figures 2, 3, Table 2) argues for the usefulness of a geographic/demographic approach. At the zonal level, the presence of a region of relatively high ART uptake in the western part of the Ethiopian highlands, an area with relatively low HIV prevalence [21] but with well established regional and NGO hospitals indicates the beneficial effect of better access to the health services. The stronger correlation between number of hospitals and number of patients on ART by urban than total zone populations (Table 2) further emphasizes the importance of accessibility to services in determining ART uptake. The weak but positive correlation for distance between ART health centers and hospitals and utilization (Table 2) may be due to the opposing effects of patients traditionally preferring to be treated at hospitals rather than the usually nearer health centers and transportation constraints [20], and lower knowledge about the availability of hospital ART services in rural areas [27]. Many of the towns with regional referral hospitals (Figure 1) and mission or secular NGO hospitals had the highest population-based utilization rates. Contributing factors appear to be the capacity of these relatively better staffed and equipped ART sites, their location on major transportation routes, and associated influx of patients from other communities [18,20,48]. These data indicate that ART site selection, catchment area delineation, and patient referral and follow-up programs will have to consider these spatial and demographic factors, as well as the shallow distance decay curve associated with the health seeking behavior of severe ill patients [20,49]. The disparate distribution of ART sites within zones and regions, often near borders (figures 1, 2), will also require accommodations in catchment area delineation and interregional patient referral, as pointed out by the MOH [6].

These relationships require further studies that consider HIV infection rates, more recent demographic data, and information on patients' place of residence. Although the ART data presented here are thought to be reasonably complete, deficiencies in the monitoring and reporting system [11,50] and the start-up of some hospitals and all health centers during 2006 point out the need for follow-up studies, especially among ART facilities with low capacity.

The population-based ART utilization rates presented in this preliminary study can provide only broad estimates of coverage for towns with treatment facilities and for the zones and regions. Their major weaknesses are the population data projected by the 1994 census and the lack of information on the completeness of the ART data. Future studies may also benefit from more representative HIV data for communities [12,27,48] and mobile populations [51,52] because they influence the demand for treatment and thus the number of AIDS patients seen at VCT centers [53] and probably also at ART sites. Information on patients' place of residence and mobility, important for patient referral and follow-up [6], may have to be gathered during treatment in view of the above mentioned elimination of the proof of residency requirement during registration for ART.

## Conclusion

We conclude that the sharp increase in ART utilization in 2006 is largely due to a combination of the provision of free treatment at a rapidly increasing number of sites. The present study provides a preliminary assessment of the rapidly improving ART accessibility and utilization situation, which calls for follow-up studies examining a larger number of push and pull factors using more recent ART, epidemiological, demographic and behavioral data. Several barriers to scaling up ART utilization are being addressed by the MOH, which is planning to carry out operational research with WHO support to identify the causes of poor treatment adherence and to strengthen the referral and linkage systems toward better service uptake. It will also be necessary to accelerate the decentralization of ART to improve access to treatment in the rural areas and particularly in the peripheral regional and zones, and to improve the treatment and care environment. This will require the improvement of working conditions and training opportunities for health workers, continued expansion of the health center construction program and the training program for mid-level health workers, the promotion of community and home-based care as part of comprehensive care programs, and the reduction of HIV-related stigma through multiple modern and traditional media targeted at all social and cultural groups.

## Competing interests

Dr. Yibeltal Assefa is the Coordinator of the Health Sector Response of the HIV/AIDS Program of the Federal Ministry of Health, Addis Ababa, involving coordinating the collection and management of the national ART data.

## Authors' contributions

HK conceived and designed the study, carried out the data analysis and prepared the drafts. YA is in charge of collecting and managing the national ART data at the Ministry of Health and helped with the location of ART sites and with the writing and revising of the manuscript. AA prepared all maps and assisted with revising the manuscript. MSM and DHM helped with drafting and revising the manuscript and identified source material. All authors read and approved the final manuscript.
